# Epigenetic domains found in mouse embryonic stem cells via a hidden Markov model

**DOI:** 10.1186/1471-2105-11-557

**Published:** 2010-11-12

**Authors:** Jessica L Larson, Guo-Cheng Yuan

**Affiliations:** 1 Department of Biostatistics, Harvard School of Public Health, Boston, Massachusetts, USA; 2 Department of Biostatistics and Computational Biology, Dana-Farber Cancer Institute, Boston, Massachusetts, USA

## Abstract

**Background:**

Epigenetics is an important layer of transcriptional control necessary for cell-type specific gene regulation. Recent studies have shown significant epigenetic patterns associated with developmental stages and diseases. However, previous studies have been mostly limited to focal epigenetic patterns, whereas methods for analyzing large-scale organizations are still lacking.

**Results:**

We developed a hidden Markov model (HMM) approach for detecting the types and locations of epigenetic domains from multiple histone modifications. We used this method to analyze a published ChIP-seq dataset of five histone modification marks (H3K4me2, H3K4me3, H3K27me3, H3K9me3, and H3K36me3) in mouse embryonic stem (ES) cells. We identified three types of domains, corresponding to active, non-active, and null states. In total, our three-state HMM identified 258 domains in the mouse genome containing 9.6 genes on average. These domains were validated by a number of criteria. The largest domains correspond to olfactory receptor (OR) gene clusters. Each *Hox *gene cluster also forms a separate epigenetic domain. We found that each type of domain is associated with distinct biological functions and structural changes during early cell differentiation.

**Conclusions:**

The HMM approach successfully detects domains of consistent epigenetic patterns from ChIP-seq data, providing new insights into the role of epigenetics in long-range gene regulation.

## Background

Well before the first eukaryotic genome was sequenced, the notion that chromatin is partitioned into larger than gene-size domains (such as the heterochromatin and euchromatin) was conceived [1,2]. Genes that are functionally related and co-regulated are often located close to each other. These include the *Hox *and the *β-globin *gene clusters [3,4]. More generally, unrelated spatially proximal genes can still be co-regulated, and such co-regulation can in part be explained by long-range chromosomal interactions [5]. However, a major barrier for understanding the mechanism for long-range gene regulation is the difficulty of generating high-resolution long-range chromosomal interaction data on a genomic scale [6].

The fundamental unit of chromatin is the nucleosome, which is a histone octamer wrapping up ~147 bp DNA. The amino acid residues on the N-terminal tails of the histone proteins can be covalently modified in a number of different ways, and different biochemical modification marks may have very different biological functions [7]. In recent years, genome-wide distributions of various histone modifications in several eukaryotic organisms have been mapped using chromatin immunoprecipitation followed by either microarrays (ChIP-chip) or DNA sequencing (ChIP-seq) [8-10]. It is thought that different combinations of histone modifications result in different functional specificities [11] and that several modifications form broad domains [12-14], which is helpful in stabilizing chromatin state and transmitting the states in cell division [15]. Integration of multiple histone modification marks has identified distinct epigenetic patterns, associated gene activities, and regulatory elements [16,17]. However, most of these earlier studies are done on a gene-by-gene basis and have ignored the spatial correlation of epigenetic patterns.

Because it is difficult to detect long-range chromosomal interactions through experimental methods, it is valuable to develop computational methods to identify long-range correlations such as epigenetic domains based on histone modification data. This can be used to infer such interactions. Here we use the term "epigenetic domain" to refer to a large-scale region containing multiple genes with epigenetic patterns. For the rest of the paper, we will use the terms "epigenetic domain" and "domain" interchangeably. Such epigenetic domain methods should provide mechanistic insights into coordinated gene regulation. Several approaches have already been developed in recent years [18-24]. However, two of the studies [18,20] partition the genome without any constraint, making the results hard to interpret. Other studies consider only a single histone modification mark, which can only give partial epigenetic information [19,20]. Or the researchers only examined a certain pattern of two modifications [21,22], simply small regions [23], or were not looking at neighboring regions, only clusters as a whole [24]. Therefore, further improvement is still needed.

Here, we developed a novel method that uses genome-wide histone modification data to identify epigenetically consistent, multi-gene domains. We applied our method to analyze a recently published ChIP-seq dataset for mouse embryonic stem (ES) cells [10,25] and found that we were able to identify a number of domains that are significantly large (i.e. not just due to chance). We validated our predictions by integrating a number of data sources. We also explored these histone modifications in the neural progenitor (NP) cell line to determine what, if any, changes in domain size, structure, and/or function occur during early cell differentiation. Our method provides a useful tool to investigate the role of epigenetic domains in development.

## Results and Discussion

### Data type, preliminary manipulation and clustering

**Figure 1:**
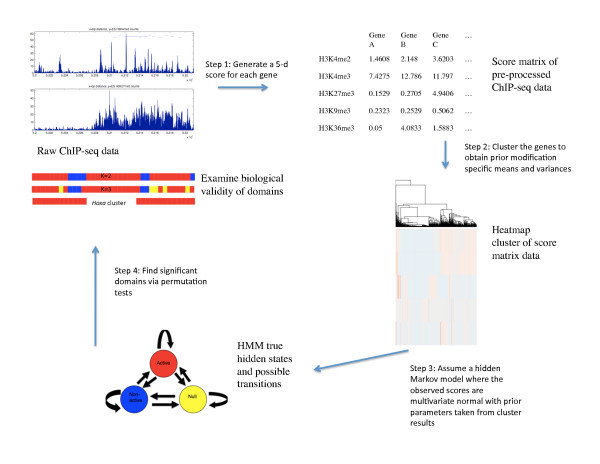
**Outline of methods. **Brief four-step depiction of methods used in this study to find chromatin domains

Genome-wide location data for five different histone modification marks, H3K4me2, H3K4me3, H3K27me3, H3K9me3, and H3K36me3, was taken from two ChIP-seq datasets [10,25]. Since our goal was to identify multi-gene regions with consistent histone modification patterns, we treated each gene as a unit and summarized the local distribution of each histone modification by a single score. We arrived at a five-dimensional summary score for each gene, corresponding to the average of the sequence tag counts over the promoter (for H3K4me2, H3K4me3, H3K9me3, and H3K27me3) or coding region (for H3K36me3) (Figure 1).

**Figure 2:**
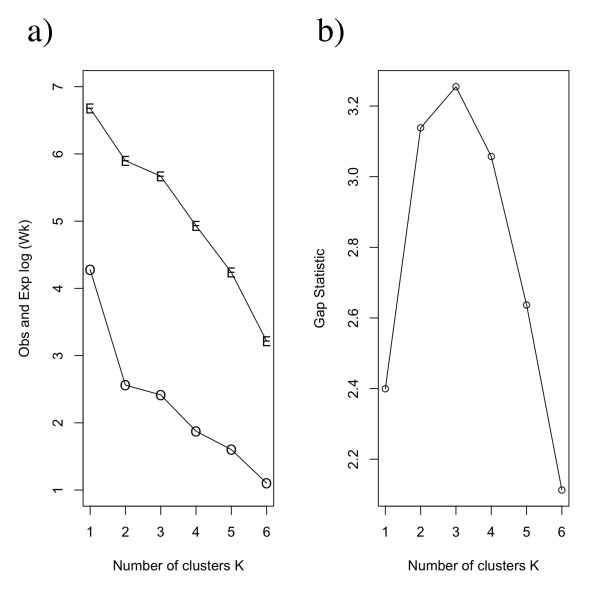
**Results from gap statistics analysis of a random sample of the data. **The expected and observed *log (W_K_) *values are shown in (a) for various levels of *K *(the number of clusters), where *W_K _*is the pooled within cluster sum of squares around the cluster means. The number of clusters versus *Gap(K)*, the difference between the observed and expected values (mean value for 1000 random bootstrap permutations), is shown in (b). According to these results, three is the optional number of clusters for our data.

We clustered the genes based on their histone modification patterns and determined the number of clusters via calculations of a "gap" statistic [26], which compares the observed within-cluster dispersion for one run with that expected by chance (i.e. averaging over 1000 permutations). The maximum gap value is achieved at K = 3, where K is the number of clusters, and the two-cluster partition also corresponds to a relatively high gap statistic (Figure 2). In the two-cluster scenario, there is a cluster of genes associated with high H3K4me2 and H3K4me3 levels; while the other is associated with moderate H3K27me3 level. In the three-cluster scenario, there is an additional cluster characterized by moderate level of the other modifications.

### Prediction of epigenetic domains

To constrain on spatial correlation of histone modification patterns, we applied a hidden Markov model (HMM) to partition the genome into epigenetic domains based upon the five-dimensional summary histone modification scores. In accordance with previous methods [18], we assumed that the emission probability can be approximated by multivariate Gaussian distribution. This gives a better fit than a Poisson distribution (Additional file 1: Figure S1).

For hidden-state assignment, we compared the results from two commonly used methods: the Viterbi and Forward-Backward algorithms, on Ch19. The results from the methods were 99.8% identical (Additional file 2: Figure S2). Since the Viterbi method is more computationally efficient and essentially as accurate, it was used to assign our hidden gene states.

We considered two model configurations, corresponding to two and three hidden states. For the rest of this paper, we will further explore these two choices and compare the corresponding results.

**Figure 3:**
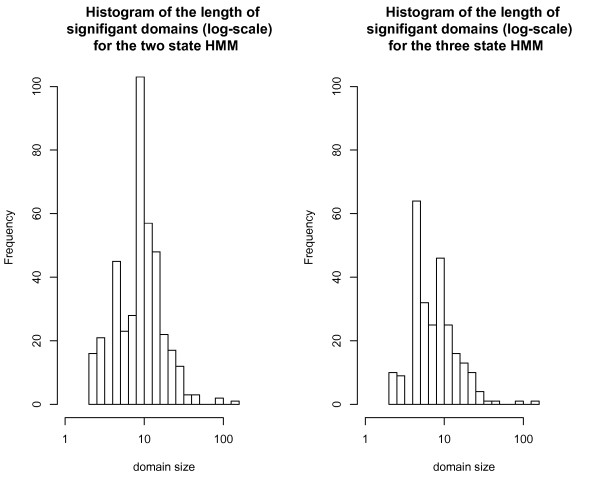
**Histogram distribution of significant domains. **Distribution of significant domains for the (a) two-state HMM and (b) three-state HMM. X-axis corresponds to the domain size; y-axis corresponds to frequency of observation.

We used a likelihood ratio test to evaluate the statistical significance of a detected domain. With a false discovery rate (FDR) cutoff of 0.01, we found 401 and 258 significant domains based on their likelihood ratio test statistics for the two- and three-state HMMs respectively (Tables 1 and 2, Additional file 3: Table S1, Additional file 4: Table S2, Additional file 5: Table S3, Additional file 6: Table S4, and Additional file 7: Table S5). The average size of each domain was 11.31 genes for the two-state model and 9.66 genes for the three-state model (Figure 3). Both models found domains over the length of 155 genes and had a minimum domain size of 2.

**Table 1:** Summary statistics for significant domains in the two-state HMM

State	Number of significant domains	Min domain size	Max domain size	Average domain size	Variance in domain size
Non-active	230	2	157	14.5	186.14
Active	171	2	29	7.02	16.45
**Total**	**401**	**2**	**157**	**11.31**	**127.28**

**Table 2:** Summary statistics for significant domains in the three-state HMM

State	Number of significant domains	Min domain size	Max domain size	Average domain size	Variance in domain size
Non-active	25	2	17	9.48	12.59
Active	80	2	15	7.03	11.41
Null	153	2	155	11.06	227.02
**Total**	**258**	**2**	**155**	**9.66**	**142.28**

As expected, the two-state HMM resulted in an active state (high H3K4me2/3 and low H3K27me3 activity) and a non-active state (low H3K4me2/3 and high H3K27me3 activity) (Table 3). For the three-state HMM, we also identified three distinct epigenetic patterns: an active cluster characterized by high H3K4me2/me3 and low H3K27me3 level, a non-active cluster characterized by high H3K27me3 and moderately low H3K4me2/me3 level, and a null cluster characterized by low level of all histone marks (Table 4). This null state cannot be captured by the two-state HMM.

**Table 3:** Average (variance) histone modification activity within a state for the two-state HMM

Modification	Non-active State	Active State
H3K4me2	1.453 (2.492)	4.653 (4.836)
H3K4me3	3.580 (18.538)	14.586 (60.923)
H3K27me3	2.089 (7.346)	0.740 (0.209)
H3K9me3	0.416 (0.112)	0.398 (0.0297)
H3K36me3	0.352 (0.176)	1.685 (5.266)

**Table 4:** Average (variance) histone modification activity within a state for the three-state HMM

Modification	Non-active State	Null State	Active State
H3K4me2	2.844 (2.106)	0.216 (0.083)	4.523 (4.934)
H3K4me3	7.020 (20.585)	0.499 (0.186)	14.205 (62.267)
H3K27me3	3.623 (10.534)	0.743 (0.412)	0.695 (0.130)
H3K9me3	0.458 (0.194)	0.383 (0.038)	0.394 (0.027)
H3K36me3	0.391(0.241)	0.367 (0.190)	1.615 (5.232)

### Functional coherence of the predicted domains

It is intrinsically difficult to define a 'gold-standard' set of epigenetic domains. To test whether our predicted domains are biologically meaningful, we examined a number of properties that are associated with chromatin domains, as explained below.

**Figure 4:**
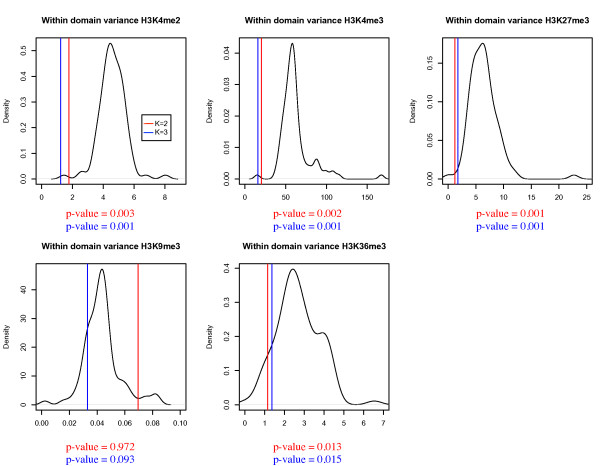
**Average within-domain variance of a modification versus a random distribution. **Two-state model (K = 2) results (and corresponding permutation test p-values) are shown in red, three-state in blue (K = 3), random distribution shown in black. For four of the modifications, the HMM domains have a significantly lower average variance (H3K4me2, H3K4me3 & H3K27me3: p-values < 0.001), suggesting that the HMM has produced coherent domain bounds.

First, we tested whether the histone modification patterns were indeed consistent within each predicted significant domain. To this end, we calculated the within-domain variance of the summary score for each modification in our significant domains, and tested whether this is significantly lower than expected by chance. Using permutation tests, we found that both the two- and three-state HMMs had significantly lower variances in the histone modifications considered in this study (p-values < 0.05), with the exception of H3K9me3 (Figure 4). This suggests that H3K9me3 does not play a major role in determining our domain states.

**Figure 5:**
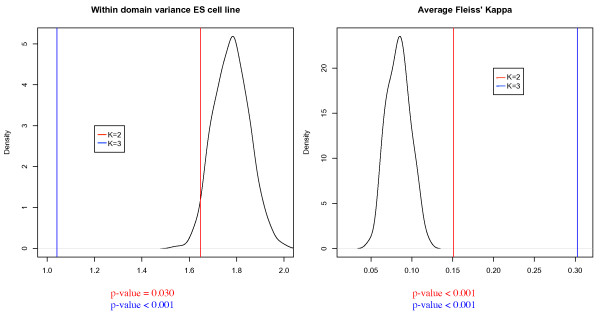
**Coherence within predicted chromatin domains. **The values (and corresponding permutation test p-values) for predicted chromatin domains (red for two state model, K = 2, and blue for three, K = 3) are compared with the distribution estimated from 1000 random permutations (black). Shown is the average within domain variance in gene expression (left) and the average level of Gene Ontology accordance as measured by Fleiss' Kappa (right) under the null hypothesis of incoherent domains. Note that our models result in significantly different values.

Second, we reasoned that genes that are embedded in the same epigenetic domain are likely to be activated or repressed together. Therefore, the gene expression levels should be more correlated within an epigenetic domain than what we would expect by chance. To test this property, we compared the within-domain variance of expression within a predicted domain with that for random neighboring gene sets of the same size (in terms of number of genes). For both models, the within-domain variance in gene expression was lower than expected by chance (p-values < 0.05) (Figure 5a). Also, the variance for the three-state HMM is lower than the two-state HMM, further supporting the notion that the three-state HMM is a more appropriate model.

Third, we asked whether the genes that are embedded in the same domain tend to have similar biological functions. To this end, we examined the Gene Ontology (GO) patterns within and between domains. We used Fleiss' Kappa [27], an accordance statistic, to measure the coherence of GO terms within our significant domains. Compared to a random selection of domain bounds, our predicted significant domains had a more concordant GO structure (p-values < 0.001) for the two- and three-state models (Figure 5b). Again, the three-state HMM had a higher level of accordance, suggesting that it is a better fit of the data.

Finally, we recognized that the histone modification patterns within a predicted domain are consistent, but substantial variance still remains. As a further validation, we asked whether our HMM was expected to provide reliable predictions under such circumstances. To this end, we designed numerical simulations mimicking the parameters for the real data (Methods). For the two-state HMM, we were 98% accurate in predicting the true states when using the observed variances, and 97% accurate when using twice the observed variances (Additional file 8: Figure S3a & Additional file 9: Figure S4a). For the three-state HMM, we were 99% accurate in our simulations (Additional file 8: Figure S3b & Additional file 9: Figure S4b). This suggests that if such domain patterns actually exist in the data, our model would be sufficiently able to detect them.

### Validation against known domains

**Figure 6:**
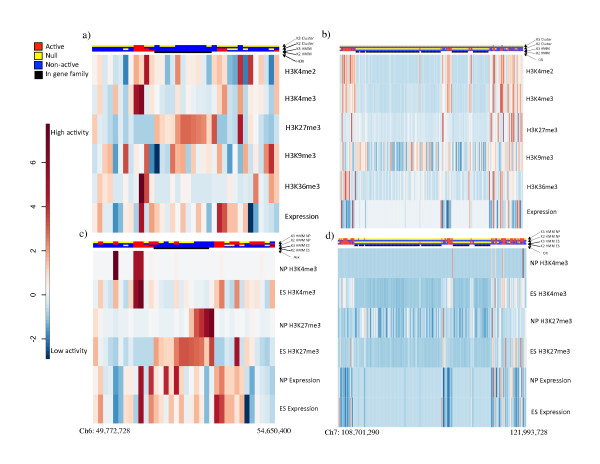
**Heatmaps of known gene clusters. **The 35 gene region on Ch6 from *Npy *to *2410066E13Rik *(49,772,728 to 54,650,400) as depictured as a heatmap of histone modification and gene expression for (a) the ES cell line and (c) ES and NP cell lines. The 250 gene region on Ch7 from *Art2a *to *Insc *(108,701,290-121,993,728) as depictured as a heatmap of histone modification and gene expression for (b) the ES cell line and (d) ES and NP cell lines. For all figures on the left (a and c), cluster state assignment is given in the first and second tracks and HMM state assignments are in the third and fourth tracks (red for active state, blue for non-active state, yellow for null state). For figures on the right (b and d), NP HMM state assignment is given in the first and second tracks and ES HMM state assignments are in the third and fourth tracks. Whether (black) or not (white) a gene is a respective gene cluster in shown in the bottom track in all figures.

The *Hox *gene clusters are a well-described epigenetic domain family. These genes regulate the anterior-posterior axis of metazoan organisms and are expressed in a sequential order during cell differentiation. In ES cells, the *Hox *genes are targeted by the Polycomb group (PcG) proteins and associated with bivalent domains [12,28]. Our method correctly detected each of the four *Hox *clusters to be in a non-active and significant domain. The results for the *Hoxa *cluster on chromosome (Ch) 6 are shown in Figure 6a. Simple K-means clustering failed to capture these state assignments.

Surprisingly, six of the ten largest predicted domains in each model are null domains enriched with olfactory receptor (OR) genes. For example, the largest OR gene cluster on Ch 7 (as described by [29]), also showed a clear distinction between HMM assignments and gene function (Figure 6b). That is, all the 208 OR genes in this 250 gene region are assigned to the same domain type, while their neighboring genes (and even non-OR genes within this cluster) are found to be in different domain types. The state assignments also correspond nicely with expression level changes (Figure 6b).

The OR genes are only expressed on in sensory neurons, and only a single gene (out of 1300) is activated in each cell [30]. We searched the literature for domain-level regulation of OR genes and noticed a recent paper showing that the selectivity of OR gene activation is established by the long-range chromosomal interaction between a single enhancer element and its target promoter [31]. Due to the lack of sequence specificity of such an interaction, it is reasonable to assume that the maintenance of an open chromatin environment over a large domain plays an important role in the regulation of OR genes. A comparison of the domain organization between sensory neurons and other cell types may provide further insights into the unique feature of OR gene regulation.

**Figure 7:**
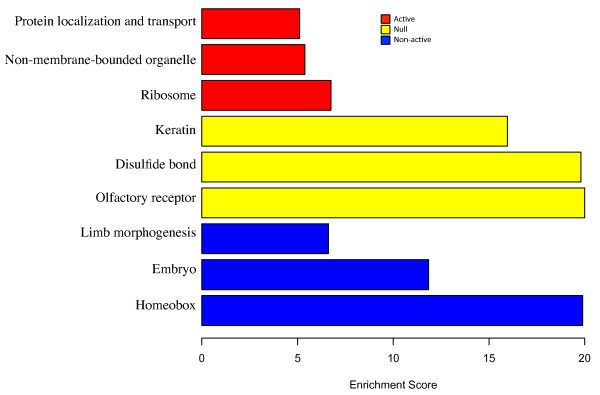
**DAVID analysis of genes within each ES significant domain. **Genes in each state are described by the top three significant DAVID clusters. Red corresponds to genes in the active state, blue for those in the non-active state and yellow for null state.

We investigated the enriched biological functions associated with each domain type by exploring the top three significant DAVID clusters [32] (Figure 7) for genes in each domain. We found that the active domains are enriched with genes involved in key cellular processes, such as protein localization and transport. In contrast, the genes embedded in null domains tend to be associated with functions of a terminally differentiated cell-type, e.g. keratin and olfactory receptors. Non-active genes tend to be involved with development, e.g. limb morphogenesis and homeobox. Thus, each domain type is characterized by a different function. Our analysis suggests that the epigenetic information provides useful insights into cell-type specific regulation.

### Domain changes in neural progenitor cells

Previous studies have identified dramatic epigenetic changes during cell differentiation [10,14,33]. To test whether the epigenetic changes also occur at the domain level, we applied our three-state HMM to infer domain states in the NP cells and compared the results for ES cells (Additional file 10: Table S6). While the overall change is moderate, we noticed some important changes at specific loci. For example, we found that the non-active *Hoxa *domain shrank significantly in NP cells (Figure 6c), consistent with a previous time-course study [34]. Such change is accompanied by activation of certain *Hoxa *genes in NP cells (Additional file 11: Figure S5a). The loss of H3K27me3 is accompanied by a moderate increase of H3K4me3 and expression levels. Little changed for the OR genes between the ES and NP cell lines (Figure 6d and Additional file 11: Figure S5b).

In total, we found that 179 of the 258 domains in ES cells contain at least one gene that changes epigenetic state in the NP cell line. For each significant ES domain, if any genes within the domain have a new state in the NP cell line, then those genes would represent a domain change. Note that there may be smaller NP domains than their corresponding ES domains, and that one ES domain could be multiple NP domains. Thus our 258 significant ES domains were 450 NP domains (Additional File 10: Table S6). For these NP domains changes, we again used DAVID to analyze the functions of the genes that change state during this early stage of development by examining the top three significant DAVID clusters (Additional file 12: Figure S6) for each of our 6 types of change (and for genes that remained state unchanged). Eight domains are non-active in ES cells but become active in NP cells. These domains are enriched with developmental regulators. On the other hand, six domains, containing 108 genes, switch from null to active states during differentiation.

Some of the non-active domains in ES cells remain non-active in NP cells, and they may be important for further development. On the other hand, a number of domains switch from the active to the non-active state in NP cells, and the functions of these domains are typically related to early embryonic development. They are enriched with functions such as sex differentiation and apoptosis. Thus, the early developmental genes are epigenetically marked in ES cells rather than at a later developmental stage.

## Conclusions

As the nucleosome level chromatin states become increasingly well described [15], the next frontier becomes the characterization of higher-order chromatin structure. Numerous studies have suggested that epigenetic domains play important roles in gene regulation [5,35], yet the detection of genome-wide long-range chromosomal correlations remains technically challenging [6]. On the other hand, genome-wide histone modification data provides important information about long-range gene regulation [12-14,36]. Thus it is valuable to develop computational methods to detect large-scale domains based on histone modification data.

Here we developed an HMM-based method to predict epigenetic domains. A similar method has recently been used to characterize the epigenetic states associated with gene promoters [37]. However, we extend this approach to identify large-scale epigenetic patterns. Compared to previous domain detection methods [18-20], our model can easily accommodate additional histone modification marks and provide easily interpretable prediction outcomes.

Our model detects three distinct types of epigenetic domains, two of which are transcriptionally inactive, which we call non-active and null. These two domain types are also distinct functionally in terms of both activation potential and biological functions. For example, both the *Hox *and OR gene clusters form epigenetic domains that are transcriptionally inactive in ES cells. Yet the *Hox *genes are critical for the overall development and are found in non-active domains in the ES cell line, whereas OR genes are expressed only in specific adult cell types and are found in null domains in the ES and NP cell lines. Therefore the epigenetic patterns provide more regulatory information than can be appreciated by gene expression data alone, signifying the importance of characterizing domain types.

Recent studies have shown that spreading of histone modification marks is an important epigenetic signature of cell differentiation [13,14]. Our work can be viewed as an extension in terms of considering the combinatorial patterns of multiple histone modifications instead of focusing on a single modification alone. Indeed, we found changes of epigenetic domains from ES to NP cells, which are accompanied by coordinated activation of neuron-specific genes. Our analysis suggests that epigenetic domain-level changes may play an important role in neuron differentiation and organismal development.

We recognize that our model still has a number of limitations. For example, the reduction of the spatial epigenetic patterns by gene-level summary scores precludes us from pinpointing the exact locations of domain boundaries. In addition, we have ignored the correlation between different histone modification marks, which may important if data for a large number of marks is available. We plan to overcome these limitations in future studies.

## Methods

### Gene-level Summary Score

Gene annotation was based on Refseq; we obtained 17,772 genes in total. For four of the five modifications (H3K4me2, H3K4me3, H3K27me3 and H3K9me3), the tag counts peak near the TSS; therefore the average was taken over the regions from -2 kb to +2 kb with respect to the TSS. The tag counts for H3K36me3 are highest around the 3'-end of the coding region of a gene; thus, we took the average tag counts over this area as a gene's corresponding score. Genes with more than 50% repetitive sequences in either of these two regions were not used in further analysis. Our preliminary data manipulations led to the elimination of sites with poor ChIP-seq coverage, resulting 17,469 (98.3%) genes used in further analysis.

### Clustering

With the gene-specific summary scores, each gene is associated with an *m*-dimensional vector, where *m *is the number of histone modification marks. As an initial guess for the number of domain types, we clustered the *m*-dimensional vectors using the *k*-means average agglomeration clustering method. The optimal cluster number *k *was selected using the gap statistic [26], defined as

$$Gap(k)=E\left(\log\left(W_{k}\right)\right)-\log\left(W_{k}^{*}\right),$$

where $W_{k}^{*}$ is the observed within-cluster sum of squares around the clusters means for one run, and *E*(.) represents the mean value for 1000 random bootstrap permutations.

### Hidden Markov model

We chose HMM to infer domain locations, where the hidden state at a given gene represented the associated domain type, and the emission variables are the *m*-dimensional vector summarizing the local histone modification pattern. We assumed that our emissions followed a multivariate Gaussian distribution.

When dealing with ChIP-seq data, researchers often like to assume a Poisson distribution for the counts mapped to each bin. This was not appropriate for our analysis for two main reasons: (1) we fit our model on non-integer summary scores to examine domain structure at the gene level and (2) the multi-dimensionality of our study. By the central limit theory, even if our raw counts followed a Poisson distribution, an average score of these counts (say over a promoter region) would follow a Gaussian distribution. To evaluate all five modifications simultaneously, we assumed that together they were from a multivariate Gaussian emission distribution with no covariance structure. We also checked the validity of our assumption by comparing distribution of the score data to its corresponding Poisson and Gaussian distributions. For these reasons, we assumed that our score data followed a multivariate Gaussian distribution.

We used the expectation-maximization (EM) algorithm to estimate the model parameters, and then used the Viterbi algorithm to infer the maximum likely state configuration [38]. One technical problem is that the EM algorithm can only achieve local optimization, and the results are dependent on the initial condition. One possible approach to overcome this problem is to repeat the procedure many times, each with a randomly selected initial guess. However, we found that it is more efficient to choose a particular initial guess based on the clustering results; that is, using the cluster means and variances as initial guesses for the model parameters associated with the hidden states. To test whether our clustering method-based prior led to the optimal model (in addition to being more efficient), we compared its resulting log-likelihood to that of 100 models where we randomly selected our prior parameters from the semi-conjugate hierarchical model:

$$\begin{array}{c} \mu_{ks}|\sigma_{ks}^{2}\sim N(\theta_{k},\;s_{k}^{2})\\ \sigma_{ks}^{2}\sim Inv-\chi^{2}(\nu_{k}), \end{array}$$

where *μ_ks _*is the prior mean for modification *k *(*k = 1, 2, ..., 5*) and state *s *(*s = 1, 2, 3*), *σ_ks_^2 ^*is the initial variance for modification *k *and state *s*, *θ_k _*is the sample mean for modification *k*, *s^2^_k _*is the sample variance for modification *k*, and *ν_k _*is the degrees of freedom for modification *k *such that *E(σ_ks_^2^)=(ν_k_-2)^-1 ^= s^2^_k_*. We found that using the results from *k*-means clustering to pick our prior results in a much higher final log-likelihood than this hierarchal prior (Additional file 13: Figure S7).

We also used the Viterbi algorithm, an approximation of the Forward-Backward algorithm, to assign a state to each gene. To determine the accuracy of the Viterbi method, we compared its corresponding hidden maximum likelihood estimate (MLE) state assignment to that obtained by the forward-backward algorithm, which assigns states based on posterior probabilities, also known as a maximum a posteriori (MAP) estimate. These two algorithms were compared on the 615 gene chromosome 19 and produced similar hidden states. The Viterbi algorithm was used to assign gene state as it is more computationally efficient.

The number of hidden states was equal to the optimal number of clusters from our gap statistic results. For the mouse data considered in this paper, our mouse data, the optimal number was *K = 3*, where *K *is the number of clusters. However, the gap statistic for *K = 2 *was similar, so we compared both setups in our analysis.

### Significance of detected domains

To determine statistical significance of a domain, we first calculated a likelihood ratio test statistic for each domain *j (j = 1,2, ..., n)*:

$$\lambda_{i}(\text{X})=-2\;\ln\frac{\sup_{\Theta_{0}}L(\theta |{\text{x}}_{\text{j}})}{\sup_{\Theta }L(\theta |{\text{x}}_{\text{j}})}=-2\ln\frac{P({\text{x}}_{\text{j}}|H_{0})}{P({\text{x}}_{\text{j}}|H\;M\;M)}=-2\ln\frac{P({\text{x}}_{\text{i}}|H_{0})*P({\text{x}}_{\text{i}+1}|H_{0})...*P({\text{x}}_{\text{n}j}|H_{0})}{P({\text{x}}_{\text{i}}|H\;M\;M)*P({\text{x}}_{\text{i}+1}|H\;M\;M)...*P({\text{x}}_{\text{n}j}|H\;M\;M)},$$

where ***x***_i _is the observed *m*-dimensional vector of histone modifications for gene *i (i = 1, 2, n_j_*), *n_j _*is the number of genes in domain *j*, ***X**_i _| H_0 _~N(μ_0_, Σ_0_) *and ***X**_i _| HMM ~N(μ_s_, Σ_s_)*. Note that *μ_0 _*and *Σ_0 _*are the *m*-dimensional mean vector and diagonal variance matrix of the entire dataset whereas *μ_s _*and *Σ_s _*are their individual state-based counterparts. To calculate *λ_j_(**X**) *for each domain, the *μ_s _*and *Σ_s _*of the corresponding maximum likelihood estimate state was used. Based on likelihood theory, λ_j_(**X**) ~ χ^2 ^_df _where the degrees of freedom (*df*) is the difference in parameters between models. Thus, df = 2*S*m-2*m, where *S *is the number of states in the model. To correct for multiple hypothesis testing, the significant domains were selected such that the FDR = 0.01.

### Numerical simulations

We simulated histone modification data corresponding to prescribed domain configurations and assessed the accuracy of our model. We assumed that the histone modification data for each gene was normally distributed with the mean and variances estimated from the real data (Tables 3 &4). To further explore the model robustness against data noise, we also repeated the above simulations with variances equal to twice the observed variances. We applied our HMM inference procedure to the simulated data. The accuracy of our model was quantified as the percentage of correctly assigned states.

### Gene Ontology analysis

To test for functional coherence, we examined the accordance of GO memberships within a domain via a new statistic for each significant domain. For domain *j*, we calculated the level of GO accordance, *Y_j_*, with Fleiss' Kappa [27], which is a generalization of the standard kappa for more than two raters (or in our case, ontologies). We then calculated the average accordance for these domains, and compared it to a distribution made under the null hypothesis (i.e., the hypothesis that domains are independent of GO memberships).

For the null distribution, we took into account the fact that neighboring genes often share GO annotations. To this end, we selected 1000*n random sections of the genome (of corresponding equal length in terms of number of genes to our significant domains), calculated their *Y_j_*'s and averaged them to get a null distribution for our average accordance. The p-values were evaluated as the proportion of permuted means that are larger than the observed mean accordance. Thus the minimum possible p-value was 0.001.

### Gene expression data analysis

To account for variability in gene activity, expression data in the ES cell line was normalized across data from 23 cell lines [10,25] to get a Z-statistic for each gene. We then calculated the within-domain variance for each significant domain and compared the average of these values to that of a random permutation.

To determine the significance of our within domain variance, we randomly selected 1000*n sections of the genome (each with an equal number of genes as its corresponding significant domains) and calculated the average within domain variances.

### Epigenetic domains in NP cells

The histone modification data in NP cells were obtained from a published dataset [10,25]. The ChIP-seq data in NP cells were normalized against those in ES cells by a negative-binomial regression as recommended [39]. We assumed that the model parameters in the NP cells are identical to those in the ES cells, and inferred the hidden states in NP cells by using the Viterbi algorithm again. We determined domain changes by comparing the results in NP and ES cells. A domain is called changed if at least one gene changed state between the two cell lines.

## List of abbreviations

Ch: Chromosome; ES: embryonic stem; GO: Gene ontology; HMM: hidden Markov model; MAP: maximum a posteriori; MLE: maximum likelihood estimate; NP: neural progenitor; OR: olfactory receptor

## Authors' contributions

JL participated in the design of the study, carried out the statistical analysis, and wrote the manuscript. GY conceived of the study, participated in its design and data analysis, and helped to draft the manuscript. Both authors read and approved the final manuscript.

## Supplementary Material

Additional file 1 **Figure S1: **Histogram plots of each of our five modifications and their corresponding Poisson (red) and Gaussian (blue) approximation distributions. Click here for file

Additional file 2 **Figure S2: **Posterior distributions and Viterbi path for the 615 genes on Ch19. Posterior probabilities for each of the three epigenetic-states are shown in the top three plots. The black horizontal line corresponds to 0.5 probability. The bottom plot is the state assignment for each gene, determined by the Viterbi path. Genes colored blue were assigned state 1 (non-active) by the Viterbi algorithm, yellow were assigned state 2 (null), and red were assigned state 3 (active). Click here for file

Additional file 3 **Table S1: **Significant domains for the two-state HMM. Click here for file

Additional file 4 **Table S2: **Significant domains for the three-state HMM. Click here for file

Additional file 5 **Table S3: **Genes in significant non-active domains for the three-state HMM. Click here for file

Additional file 6 **Table S4: **Genes in significant null domains for the three-state HMM. Click here for file

Additional file 7 **Table S5: **Genes in significant active domains for the three-state HMM. Click here for file

Additional file 8 **Figure S3: **Simulation results for the (a) two- and (b) three-state HMMs. The two-state HMM captures the truth 98% of the time, while the three-state HMM captures it 99% of the time. The top two tracks are a simulated H3K4me3 and H3k27me3 count, respectively. The third track is the true state (based on a random permutation), and the fourth track is the states as predicted by our model. Click here for file

Additional file 9 **Figure S4: **Simulation results for the (a) two- and (b) three-state HMMs where the simulated modification counts are based on high variance models. The two-state HMM captures the truth 97% of the time, while the three-state HMM captures it 99% of the time. The top two tracks are a simulated H3K4me3 and H3k27me3 count, respectively. The third track is the true state (based on a random permutation), and the fourth track is the states as predicted by our model. Click here for file

Additional file 10 **Table S6: **Corresponding NP domains for the significant ES domains fit by the three-state HMM. Click here for file

Additional file 11 **Figure S5: **Heatmaps for the NP cell line. (a) The 35 gene region on Ch6 from *Npy *to *2410066E13Rik *(49,772,728 to 54,650,400) as depictured as a heatmap of histone modification and gene expression. (b) The 250 gene region on Ch7 from *Art2a *to *Insc *(108,701,290-121,993,728) as depictured as a heatmap of histone modification and gene expression. NP HMM state assignments are in the first and second tracks (red for active state, blue for low state, yellow for null state). Whether (black) or not (white) a gene is a respective gene cluster in shown in the bottom track in all figures. Click here for file

Additional file 12 **Figure S6: **DAVID cluster analysis of genes within each ES significant domain in the NP cell line. Genes in each type of change are described by the top three significant DAVID clusters. Red corresponds to genes in the active state, blue for those in the non-active state and yellow for null state, in the NP cell line. Click here for file

Additional file 13 **Figure S7: **Log likelihood results for the 100 randomly chosen priors (black) versus a prior based on K-means clustering (red). Click here for file
