# Supplementary figures and images for: Epigenetic domains found in mouse embryonic stem cells via a hidden Markov model
Source: BMC Bioinformatics. 2010 Nov 12;11:557. doi: 10.1186/1471-2105-11-557 (PMC2992069; doi:10.1186/1471-2105-11-557)

**H3K4me2**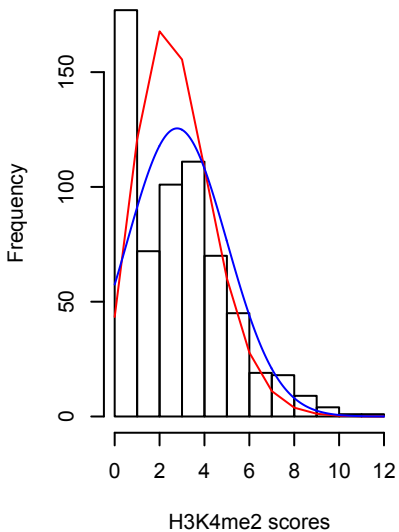**H3K4me3**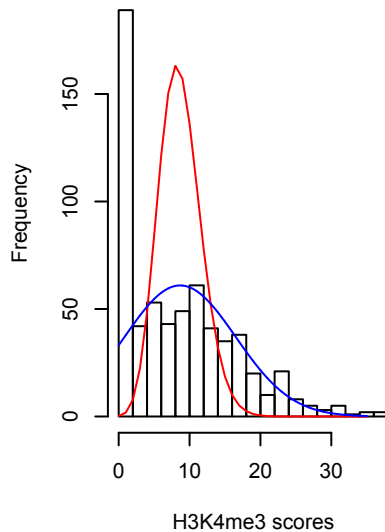**H3K27me3**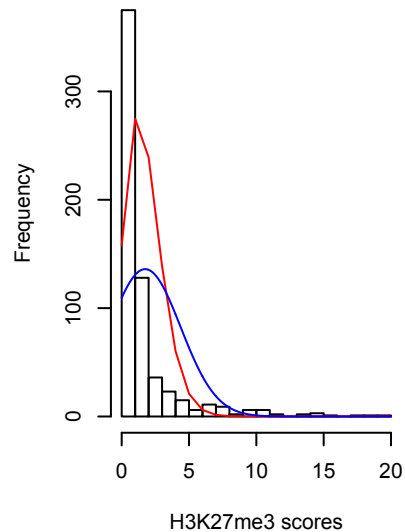**H3K9me3**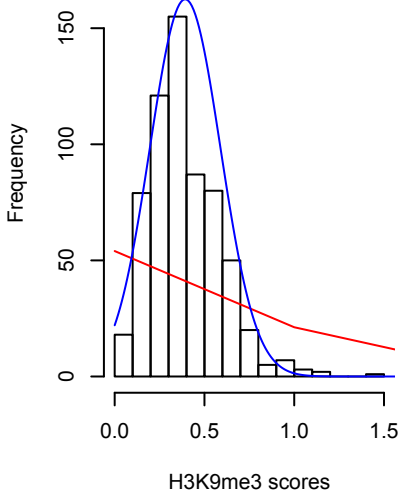**H3K36me3**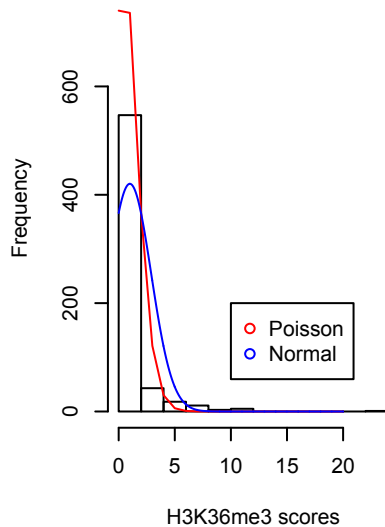

Supplement: Additional file 1 — Figure S1: Histogram plots of each of our five modifications and their corresponding Poisson (red) and Gaussian (blue) approximation distributions. [file 1471-2105-11-557-S1.PDF]

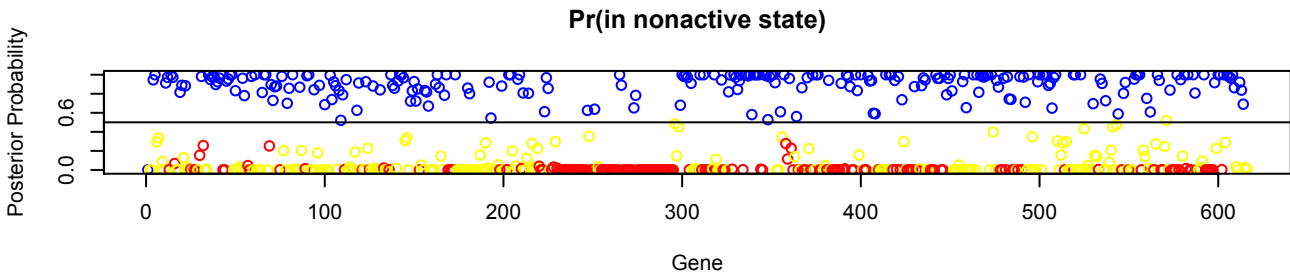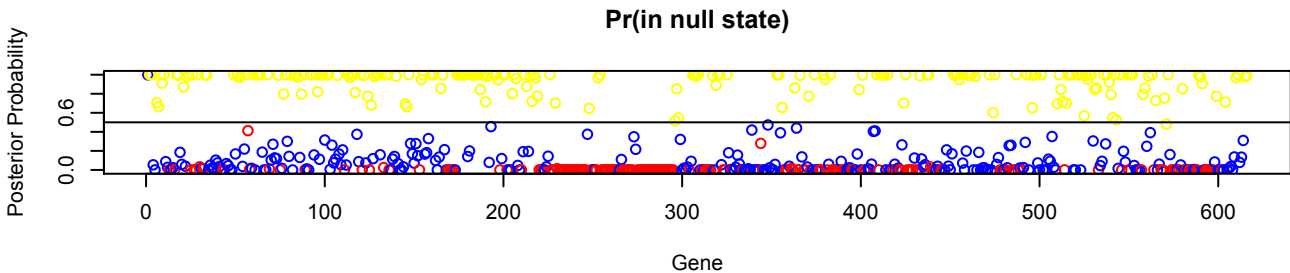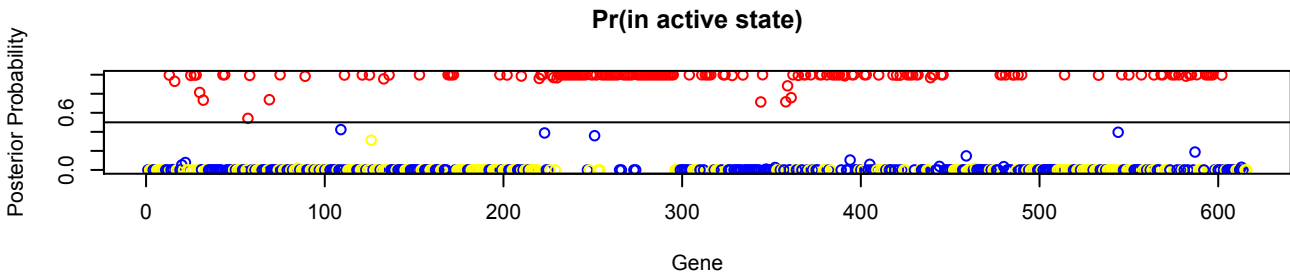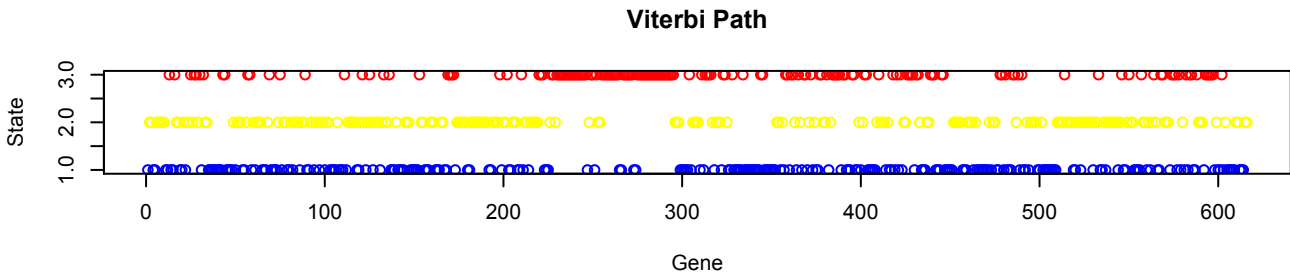

Supplement: Additional file 2 — Figure S2: Posterior distributions and Viterbi path for the 615 genes on Ch19. Posterior probabilities for each of the three epigenetic-states are shown in the top three plots. The black horizontal line corresponds to 0.5 probability. The bottom plot is the state assignment for each gene, determined by the Viterbi path. Genes colored blue were assigned state 1 (non-active) by the Viterbi algorithm, yellow were assigned state 2 (null), and red were assigned state 3 (active). [file 1471-2105-11-557-S2.PDF]

a)

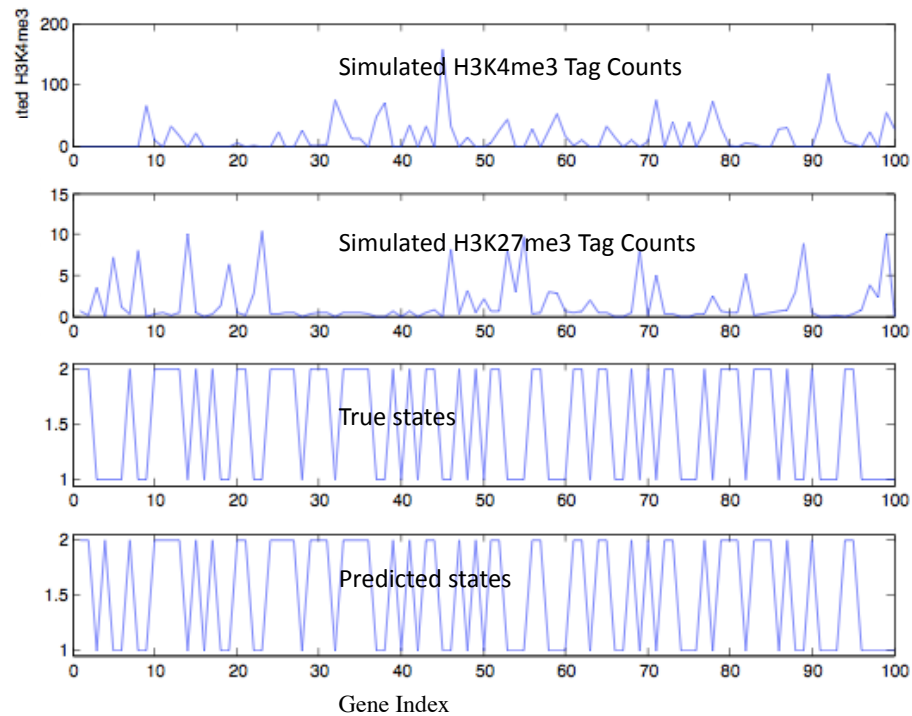

b)

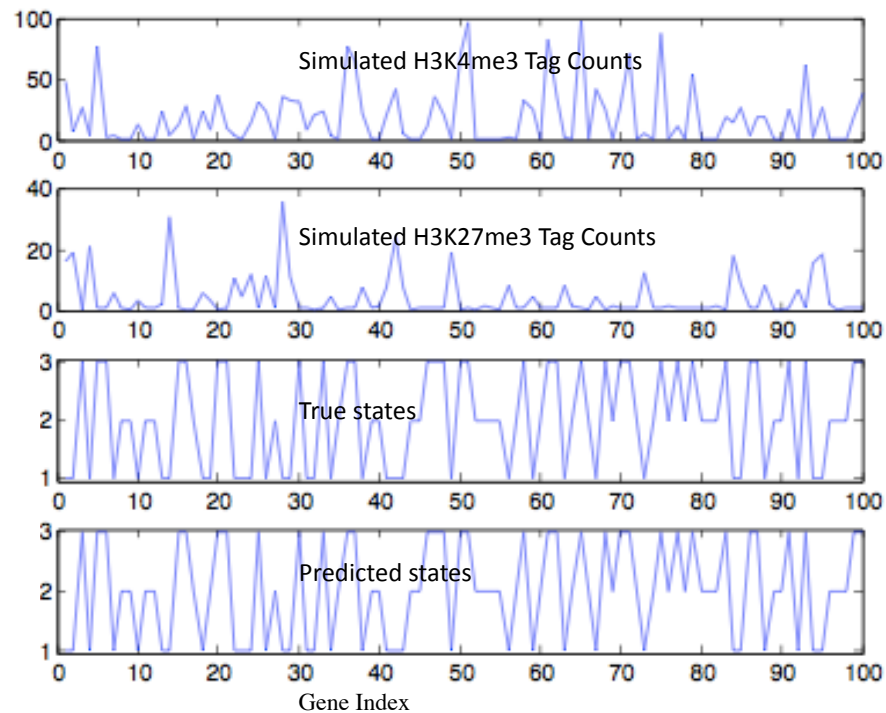

Supplement: Additional file 8 — Figure S3: Simulation results for the (a) two- and (b) three-state HMMs. The two-state HMM captures the truth 98% of the time, while the three-state HMM captures it 99% of the time. The top two tracks are a simulated H3K4me3 and H3k27me3 count, respectively. The third track is the true state (based on a random permutation), and the fourth track is the states as predicted by our model. [file 1471-2105-11-557-S8.PDF]

a)

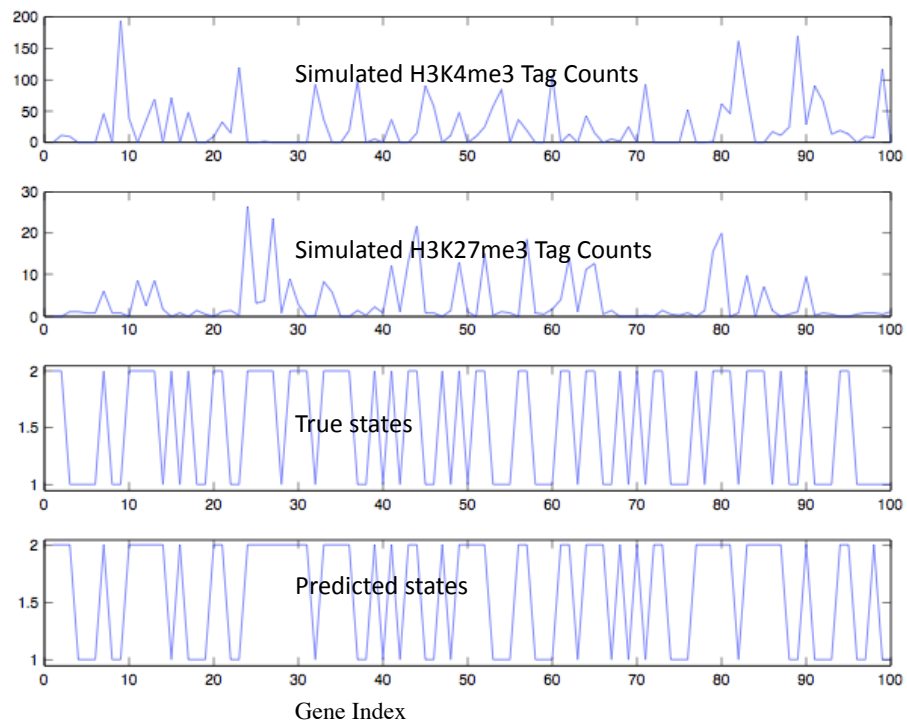

b)

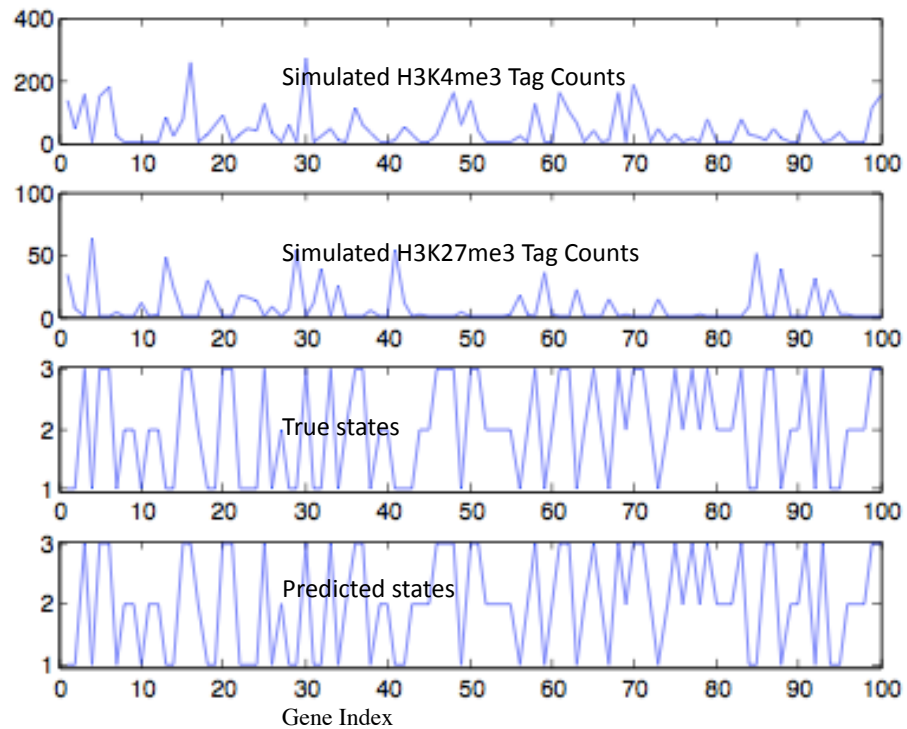

Supplement: Additional file 9 — Figure S4: Simulation results for the (a) two- and (b) three-state HMMs where the simulated modification counts are based on high variance models. The two-state HMM captures the truth 97% of the time, while the three-state HMM captures it 99% of the time. The top two tracks are a simulated H3K4me3 and H3k27me3 count, respectively. The third track is the true state (based on a random permutation), and the fourth track is the states as predicted by our model. [file 1471-2105-11-557-S9.PDF]

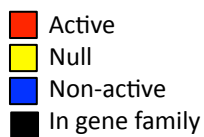

High activity

Low activity

a)

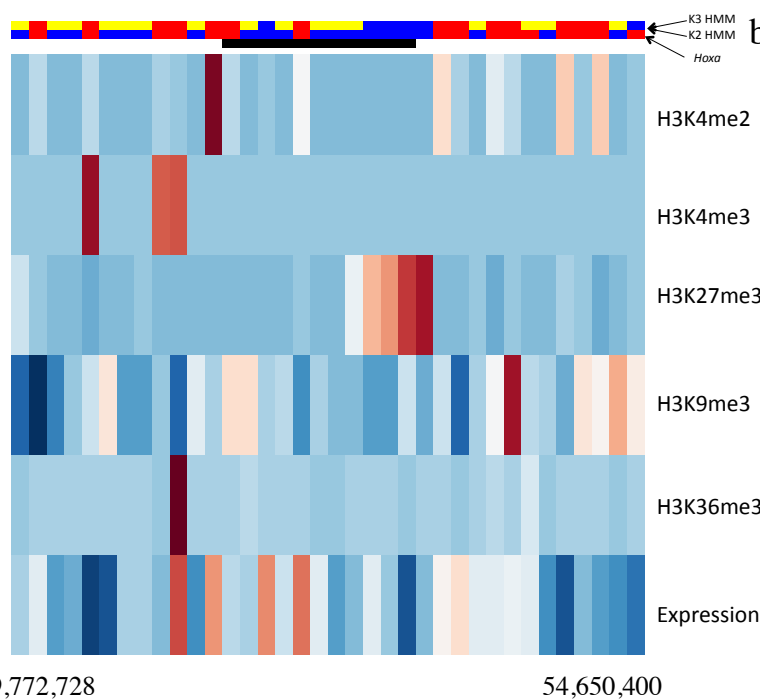

b)

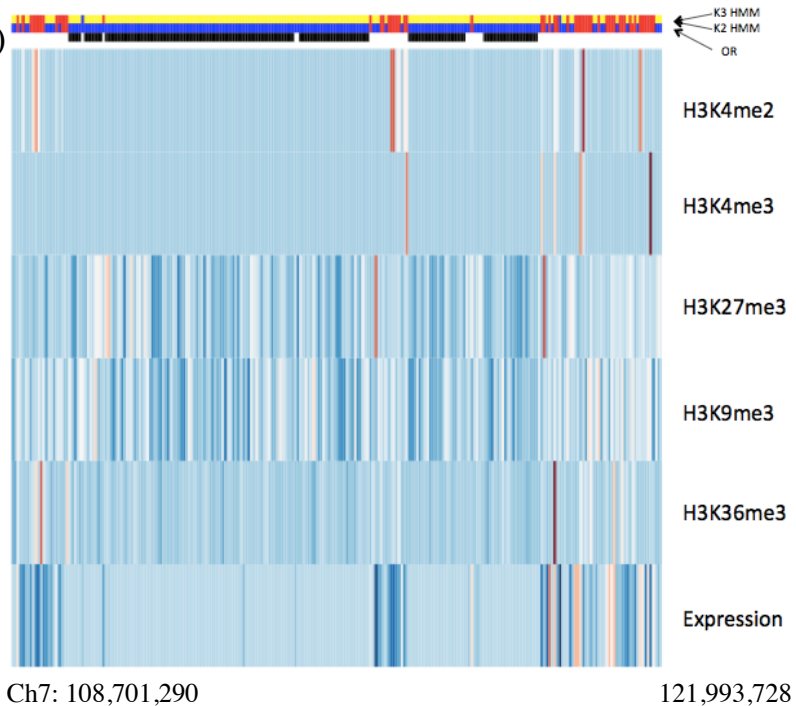

Supplement: Additional file 11 — Figure S5: Heatmaps for the NP cell line. (a) The 35 gene region on Ch6 from Npy to 2410066E13Rik (49,772,728 to 54,650,400) as depictured as a heatmap of histone modification and gene expression. (b) The 250 gene region on Ch7 from Art2a to Insc (108,701,290-121,993,728) as depictured as a heatmap of histone modification and gene expression. NP HMM state assignments are in the first and second tracks (red for active state, blue for low state, yellow for null state). Whether (black) or not (white) a gene is a respective gene cluster in shown in the bottom track in all figures. [file 1471-2105-11-557-S11.PDF]

# Log likelihood of HMM (K=3)

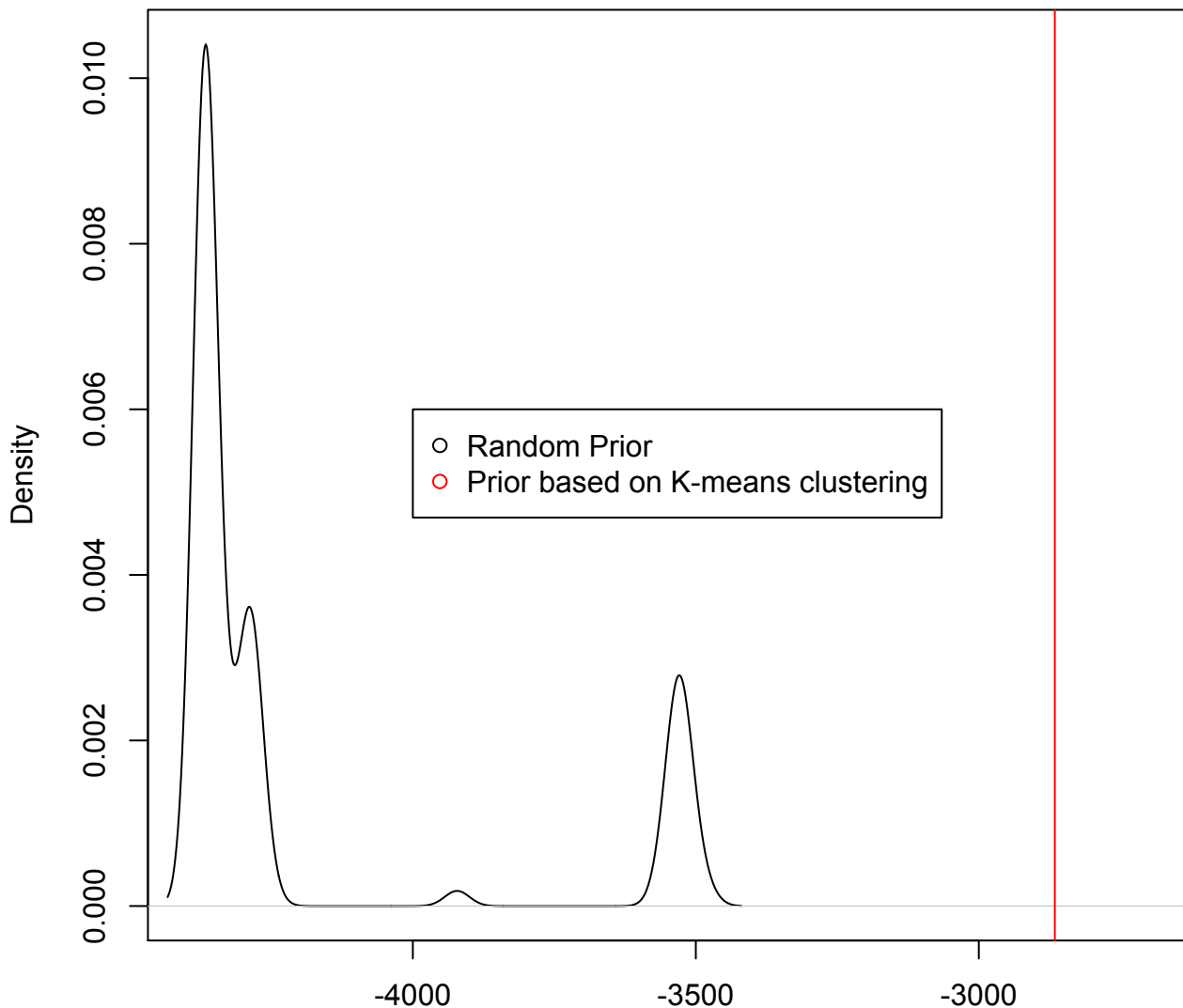

N = 100 Bandwidth = 21.77

Supplement: Additional file 13 — Figure S7: Log likelihood results for the 100 randomly chosen priors (black) versus a prior based on K-means clustering (red). [file 1471-2105-11-557-S13.PDF]
