# Supplementary material for: Epigenetic domains found in mouse embryonic stem cells via a hidden Markov model
Source: BMC Bioinformatics. 2010 Nov 12;11:557. doi: 10.1186/1471-2105-11-557 (PMC2992069; doi:10.1186/1471-2105-11-557)
Supplement: Additional file 12 — Figure S6: DAVID cluster analysis of genes within each ES significant domain in the NP cell line. Genes in each type of change are described by the top three significant DAVID clusters. Red corresponds to genes in the active state, blue for those in the non-active state and yellow for null state, in the NP cell line. [file 1471-2105-11-557-S12.PDF]

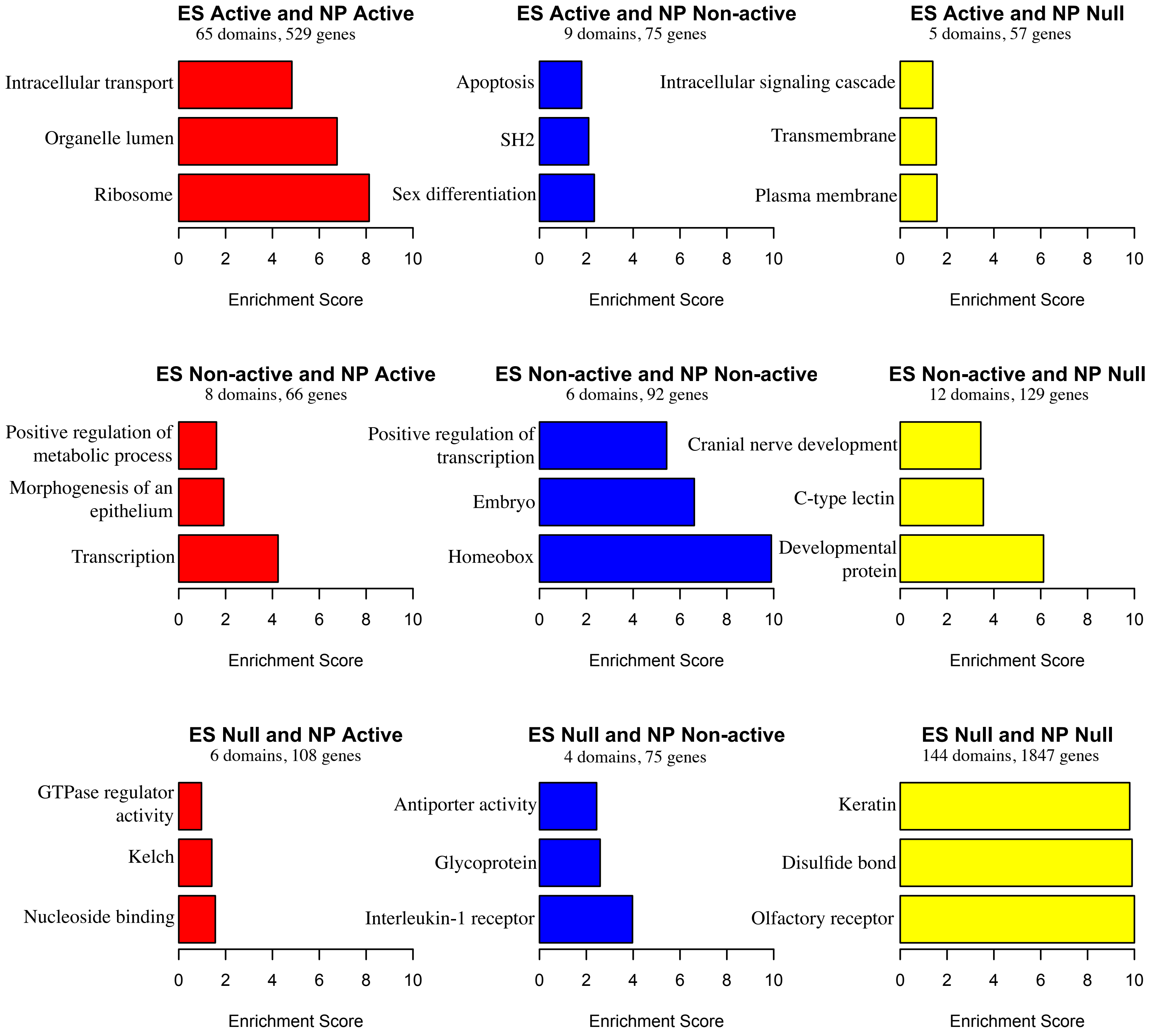
ES Active and NP Active
65 domains, 529 genes
Intracellular transport
Organelle lumen
Ribosome
Enrichment Score
ES Active and NP Non-active
9 domains, 75 genes
Apoptosis
SH2
Sex differentiation
Enrichment Score
ES Active and NP Null
5 domains, 57 genes
Intracellular signaling cascade
Transmembrane
Plasma membrane
Enrichment Score
ES Non-active and NP Active
8 domains, 66 genes
Positive regulation of metabolic process
Morphogenesis of an epithelium
Transcription
Enrichment Score
ES Non-active and NP Non-active
6 domains, 92 genes
Positive regulation of transcription
Embryo
Homeobox
Enrichment Score
ES Non-active and NP Null
12 domains, 129 genes
Cranial nerve development
C-type lectin
Developmental protein
Enrichment Score
ES Null and NP Active
6 domains, 108 genes
GTPase regulator activity
Kelch
Nucleoside binding
Enrichment Score
ES Null and NP Non-active
4 domains, 75 genes
Antiporter activity
Glycoprotein
Interleukin-1 receptor
Enrichment Score
ES Null and NP Null
144 domains, 1847 genes
Keratin
Disulfide bond
Olfactory receptor
Enrichment Score
